# Efficacy of the highly selective focal adhesion kinase inhibitor BI 853520 in adenocarcinoma xenograft models is linked to a mesenchymal tumor phenotype

**DOI:** 10.1038/s41389-018-0032-z

**Published:** 2018-02-23

**Authors:** Ulrich A. Hirt, Irene C. Waizenegger, Norbert Schweifer, Christian Haslinger, Daniel Gerlach, Jürgen Braunger, Ulrike Weyer-Czernilofsky, Heinz Stadtmüller, Ioannis Sapountzis, Gerd Bader, Andreas Zoephel, Bojan Bister, Anke Baum, Jens Quant, Norbert Kraut, Pilar Garin-Chesa, Günther R. Adolf

**Affiliations:** 1 Department of Pharmacology and Translational Research, Boehringer Ingelheim RCV GmbH & Co KG, Vienna, Austria; 2 Department of Lead Discovery, Boehringer Ingelheim RCV GmbH & Co KG, Vienna, Austria; 3 Department of Medicinal Chemistry, Boehringer Ingelheim RCV GmbH & Co KG, Vienna, Austria; 4 Department of Discovery ADME, Boehringer Ingelheim RCV GmbH & Co KG, Vienna, Austria; 5 Present Address: Boehringer Ingelheim Pharma GmbH & Co. KG, Biberach an der Riss, Germany; 6 Present Address: Boehringer Ingelheim Pharmaceuticals, Cambridge, MA 02142, USA

## Abstract

Focal adhesion kinase (FAK), a non-receptor tyrosine kinase, has attracted interest as a target for pharmacological intervention in malignant diseases. Here, we describe BI 853520, a novel ATP-competitive inhibitor distinguished by high potency and selectivity. In vitro, the compound inhibits FAK autophosphorylation in PC-3 prostate carcinoma cells with an IC_50_ of 1 nmol/L and blocks anchorage-independent proliferation of PC-3 cells with an EC_50_ of 3 nmol/L, whereas cells grown in conventional surface culture are 1000-fold less sensitive. In mice, the compound shows long half-life, high volume of distribution and high oral bioavailability; oral dosing of immunodeficient mice bearing subcutaneous PC-3 prostate adenocarcinoma xenografts resulted in rapid, long-lasting repression of FAK autophosphorylation in tumor tissue. Daily oral administration of BI 853520 to nude mice at doses of 50 mg/kg was well tolerated for prolonged periods of time. In a diverse panel of 16 subcutaneous adenocarcinoma xenograft models in nude mice, drug treatment resulted in a broad spectrum of outcomes, ranging from group median tumor growth inhibition values >100% and tumor regression in subsets of animals to complete lack of sensitivity. Biomarker analysis indicated that high sensitivity is linked to a mesenchymal tumor phenotype, initially defined by loss of E-cadherin expression and subsequently substantiated by gene set enrichment analysis. Further, we obtained microRNA expression profiles for 13 models and observed that hsa-miR-200c-3p expression is strongly correlated with efficacy (*R*^2^ = 0.889). BI 853520 is undergoing evaluation in early clinical trials.

## Introduction

Focal adhesion kinase (FAK), a non-receptor tyrosine kinase also known as protein tyrosine kinase 2 (PTK2), is only distantly related to other tyrosine kinases, with the exception of PYK2 (proline-rich tyrosine kinase 2, PTK2B). Integrating signals from integrins as well as growth factor receptors, FAK plays an essential role during mammalian development, as homozygous inactivation of the corresponding gene results in early embryonic lethality; in the adult organism, the enzyme is expressed in the majority of cell types and has been implicated in numerous physiological functions, most notably cell migration^1^.

A possible role of FAK in cancer has been investigated for a number of years, with an initial focus on tissue invasion and metastasis formation; more recently, it has been recognized that FAK may also be involved in the regulation of cancer cell proliferation and survival^2^. Consequently, FAK has attracted interest as a target for therapeutic intervention, and several ATP-competitive inhibitors with drug-like properties were synthesized and have progressed to clinical development^2,3^. However, FAK does not appear to function as an “oncogenic driver” that is activated by somatic mutations or chromosomal translocations or over-expressed due to gene amplification; although some genetic aberrations have been reported, they seem to be rare and their pathophysiological relevance is essentially unknown. Large-scale screens of cancer cell lines, an exercise frequently performed to obtain unbiased information on the potential spectrum of drug efficacy across cancer types and oncogenomes, have not been informative for FAK inhibitors. Dependence of cell proliferation on FAK signaling is generally limited to cells growing under anchorage-independent conditions, even though the enzyme may be expressed and activated in cells maintained in conventional surface culture, as judged by the autophosphorylation marker phospho-tyrosine 397. Colony-formation assays require testing of different culture conditions, i.e*.* soft agar or Matrigel^®^, which are often cell line specific and not sufficiently robust to deliver reliable quantitative readouts in large-scale screens; importantly, many cancer cell lines do not grow under these conditions.

Preclinical studies of FAK inhibitors in xenograft models of human cancer in mice have so far failed to provide reliable guidance for selection of patients who might benefit from treatment. Early clinical trials have shown that systemic inhibition of FAK is tolerated, however, efficacy signals to date have been weak, with stable disease as the best response in the majority of studies^2,3^. More recently, preclinical as well as clinical data have suggested that in mesothelioma patients, low expression of merlin, a cytoskeleton protein encoded by the tumor suppressor gene *NF2*, may be correlated with sensitivity to FAK inhibitors, however, even in this selected “merlin-low” patient subgroup stable disease was the best treatment outcome^4,5^. Further, recent preclinical data suggest that inactivation of *CDKN2A*, a gene locus encoding the tumor suppressors INK4A and ARF, may lead to FAK activation, implying that FAK inhibition may be of therapeutic value^6^.

In this report, we present the preclinical profile of a novel FAK inhibitor characterized by high potency and selectivity, notably also against PYK2, and thus suitable as a precision tool for further exploration of the pathophysiological role of FAK in cancer. Importantly, in an in vivo screening program comprising a variety of nude mouse xenograft models of human adenocarcinomas, high efficacy of BI 853520 was associated with a mesenchymal phenotype of the tumor cells, and epithelial–mesenchymal transition (EMT) markers expressed at protein or RNA level may therefore serve as actionable biomarkers of drug sensitivity.

## Results

### Potency and selectivity of BI 853520 in biochemical assays

The 2-Anilino-4-benzylaminopyrimidine has been identified as an ATP competitive FAK inhibitor scaffold^7^. X-ray analysis reveals that the N-H of the aniline and the lone pair of the pyrimidine nitrogen in position 1 form strong H-bonds to the hinge region of the kinase. Interestingly, the N/O exchange in position 4 is tolerated by FAK. The resulting 2-Aminiophenyl-4-phenoxypyrimidines show a unique binding mode which seems to be less tolerated in other kinases resulting in exquisite kinase selectivity. Further x-ray guided decoration of this privileged scaffold improved activity as well as ADME properties finally leading to BI 853520 as the clinical development compound.

In enzymatic assays using a DELFIA format, BI 853520 potently inhibited recombinant FAK (median IC_50_  = 1 nmol/L), whereas the closely related kinase PYK2 was not inhibited even at 50,000 nmol/L (Table 1). For comparison, PF-562,271, a FAK inhibitor in clinical development, showed high potency on FAK but only tenfold lower potency on PYK2 (2 nmol/L and 22 nmol/L, respectively), in agreement with published data^8^.

**Table 1:** Kinase activity and selectivity of BI 853520

	BI 853520	PF-562,271
Kinase IC_50_ [nM]		
PTK2/FAK (DELFIA^®^)	1	2
PTK2/FAK (Z-'LYTE^®^)	38.1	29.7
Selectivity (number of kinases hit ≤50% control at 1000 nM/total kinases)		
	4/262	15/37
Kinase IC_50_ [nM]		
PTK2B/PYK2 (DELFIA^®^)	>50,000	22
PTK2B/PYK2 (Z’-LYTE^®^)	2000	48
FER (Z'-LYTE^®^)	903	n.d.
FES (Z'-LYTE^®^)	1040	n.d.
PAK7 (Z'-LYTE^®^)	>10 000	n.d.

Overview of in vitro kinase activity and selectivity of BI 853520 and PF-562,271. Two different assay formats were used to determine IC_50_ values for inhibition of FAK and PYK2. Selectivity was determined on a larger kinase panel at a concentrations of 1000 nmol/L, for kinase hits from this screen IC_50_ values were measured for BI 853520

*n.d.* not determined

Further selectivity tests were performed using FRET technology. IC_50_ values for FAK and PYK2 in these assays were 38 and 2000 nmol/L, respectively (PF-562,271: 30 nmol/L and 48 nmol/L, respectively). FRET assays were then used to screen a collection of 262 additional kinases at a fixed BI 853520 concentration of 1000 nmol/L, and IC_50_ values were subsequently determined for kinases that were inhibited by at least 50%. FER and FES were the most sensitive kinases in this panel (IC_50_ = 900 nmol/L and 1040 nmol/L, respectively).

### Target inhibition and anti-proliferative activity

The human cell line PC-3, derived from a castration-resistant prostate carcinoma, was initially used to determine the cellular activity of BI 853520. Target inhibition was monitored by quantifying the concentration of FAK phosphorylated at the auto-phosphorylation site tyrosine 397 using a cell-based ELISA. Treatment with BI 853520 for 2 h resulted in a concentration-dependent reduction of the signal with a median EC_50_ value of 1 nmol/L (PF-562,271: 25 nmol/L) (Table 2). Clonogenic assays for anchorage-independent growth of PC-3 cells in soft agar showed potent inhibition of colony formation with a median EC_50_ value of 3 nmol/L (PF-562,271: 42 nmol/L); in contrast, cells grown as adherent monolayers were insensitive to BI 853520 (EC_50_ > 3 µmol/L). These results corroborate the high potency and selectivity of the compound observed in biochemical assays.

**Table 2:** Cellular activity of BI 853520

	BI 853520	PF-562,271
PD biomarker EC_50_ [nM]		
p-FAK Y397 (PC-3)	1	25
Proliferation EC_50_ [nM]		
PC-3	>3 000	1 646
Colony formation EC_50_ [nM]		
PC-3	3	42

Overview of the cellular activity of BI 853520 and PF-562,271 in the prostate cancer cell line PC-3. Inhibition of FAK autophosphorylation at site Y397 upon treatment of PC-3 human prostate cancer cells with BI 853520 and PF-562,271 was determined using an ELISA. The anti-proliferative effect of both compounds in PC-3 cells was determined on cells grown as adherent monolayers and in soft agar cultures. Metabolic activity of cells was measured using alamarBlue^®^

Although our results confirm earlier reports indicating that FAK blockade may result in inhibition of cell proliferation only in “3D” culture, we nevertheless screened a diverse panel of 243 cancer cell lines for sensitivity to BI 853520 in conventional “2D” culture. Of these, the vast majority was not affected at concentrations as high as 1 µmol/L. Interpretable sigmoid dose-response curves were obtained for few cell lines, including PC-3, but maximal inhibition was generally only about 20–30% with EC_50_ values ranging from 3 to 30 nmol/L (PC-3: 20% inhibition, 10 nmol/L); maximal inhibition was observed in Calu-6 lung adenocarcinoma cells (40%; EC_50_ = 3 nmol/L) (Supplement Table 1). Although this large-scale screen thus did not provide guidance for selection of drug-sensitive types of carcinomas we noted that cell lines derived from glioblastomas and sarcomas were over-represented in the panel of partially sensitive cell lines, a finding that will be followed up separately.

### Pharmacokinetic and pharmacodynamic parameters in mice

Following administration of a single intravenous dose of 5 mg/kg to female BomTac:NMRI-*Foxn1*^nu^ nude mice the compound showed a clearance of 36 mL/min/kg, a high volume of distribution (6.6 L/kg) and a mean residence time of 3 h. Plasma concentrations determined after oral administration of a single dose of 50 mg/kg revealed high bioavailability (90%) with a half-life of 5 h. The AUC was calculated as 46 µmol*h and a maximal concentration of 4.4 µmol/L was reached after 2 h.

**Fig. 1:**
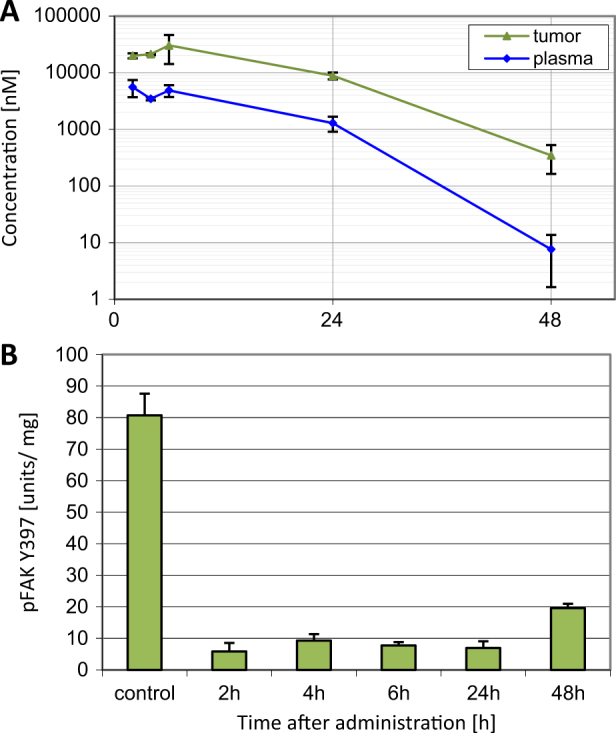
In vivo pharmacokinetic and pharmacodynamic analysis. **a** Drug concentrations in plasma or tumor of PC-3 tumor-bearing mice on day 4 (50 mg/kg BI 853520 qdx4) were determined by HPLC-MS/MS. Data represent mean values and standard error of the mean, three animals per time point. **b** Phospho-FAK Y397 expression in PC-3 tumors. Mean values and standard error of the mean, three animals per time point

To determine compound concentrations as well as pharmacodynamic activity in tumors we used BomTac:NMRI-*Foxn1*^nu^ mice xenotransplanted with PC-3 cells (Fig. 1). 50 mg/kg BI 853520 was administered orally on four consecutive days. High concentrations of BI 853520 in tumor tissues were detected already 2 h post-application on day 4, with peak levels being reached after 6 h. After 24 h, the concentration in tumor tissue was 8.9 µmol/L, whereas plasma levels had fallen to 1.3 µmol/L. Compared with untreated control tumors, phospho-FAK Y397 levels in treated tumors were strongly reduced during the entire sampling period (2–48 h).

### Efficacy and tolerability in tumor xenograft models

**Fig. 2:**
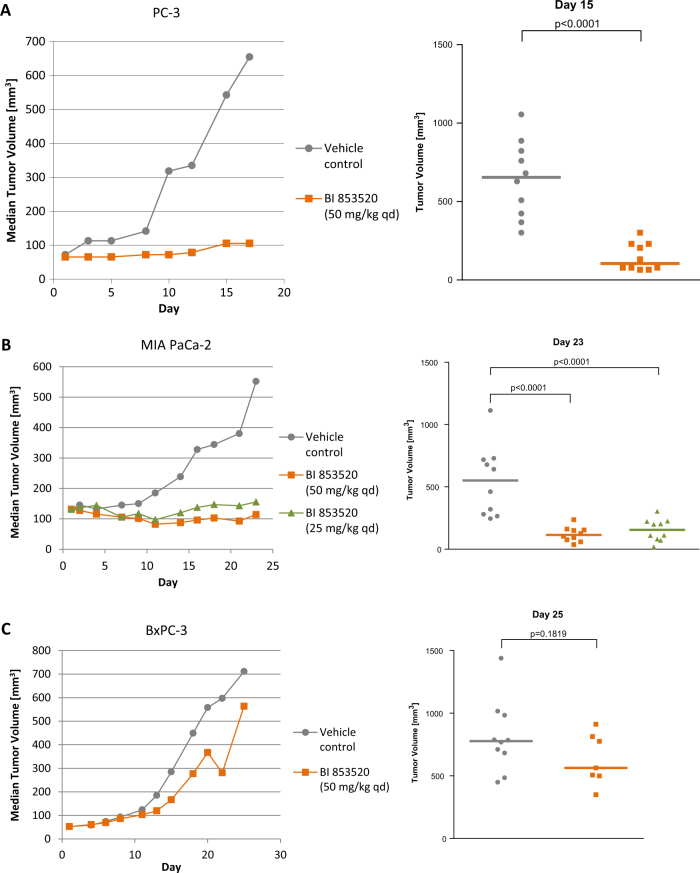
Efficacy of BI 853520 in human prostate and pancreas adenocarcinoma xenograft models in nude mice. Animals were treated with 25 or 50 mg/kg BI 853520 daily per os. Data represent median tumor volumes (left graphs) and individual as well as median values (right graph) at the given day, seven or ten animals per group. **a** PC-3 model. **b** MIA PaCa-2 model. **c** BxPC-3 model

As PC-3 prostate carcinoma cells were sensitive to growth inhibition by BI 853520 in vitro we performed a first in vivo study in this model, using tumors growing subcutaneously in nude mice. Treatment was initiated when tumors had reached diameters of 6-8 mm (seven animals per group). Daily oral doses of 50 mg/kg were well tolerated as judged by body weight gain and absence of clinical signs. Seventeen days after the start of treatment, vehicle-treated control tumors had reached a median volume of 644 mm^3^; tumor growth was significantly inhibited by BI 853520 (TGI = 93%, *p* < 0.0001, Fig. 2a).

We subsequently performed additional studies in pancreas adenocarcinoma xenograft models. In the MIA PaCa-2 model, treatment with BI 853520 at 25 mg/kg or 50 mg/kg once daily resulted in high efficacy, with TGIs of 94% and 104%, respectively, and partial regressions were observed in 5 and 6 out of 10 tumors in the respective treatment groups (Fig. 2b). In a second, independent study this result was confirmed for doses of 25 mg/kg (TGI = 96%); significant growth inhibition was also observed at 12.5 mg/kg (TGI = 89%, Supplement Fig. 1). In contrast, BI 853520 dosed at 50 mg/kg did not show statistically significant efficacy in a second model derived from human BxPC-3 cells (TGI = 29%, Fig. 2c).

**Fig. 3:**
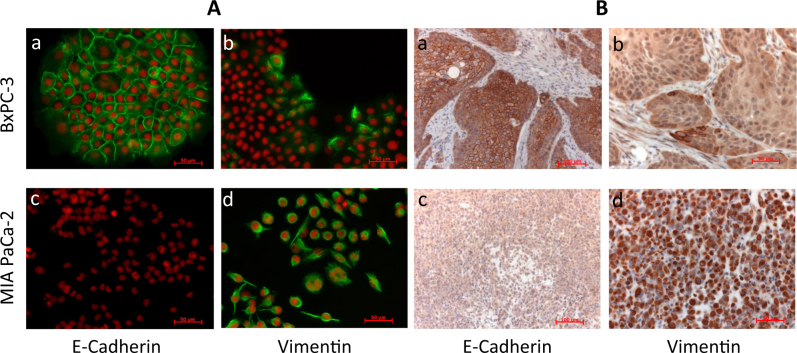
E-cadherin and vimentin expression in MIA PaCa-2 and BxPC-3 cells. **a** Adherently growing cells were fixed and stained with antibodies against E-cadherin or vimentin followed by Alexa Fluor 488 conjugated secondary antibody; nuclei were visualized by staining with propidium iodide. Size bars represent 50 µm (a–d). **b** Tumor xenografts were fixed and analyzed by immunohistochemistry for expression of E-cadherin or vimentin (brown label) and by hematoxylin and eosin (blue label). Size bars represent 100 µm (a, c) or 50 µm (b, d)

This striking difference in sensitivity prompted us to search for biomarkers of drug efficacy. We had noted previously that in tissue culture, BxPC-3 cells form well-connected epithelial-like clusters of cells, whereas MIA Paca-2 cells grow as single cells with a spindle-like or fibroblast-like morphology, lacking the cell–cell contacts characteristic of epithelial tissue and thus exhibiting a mesenchymal phenotype. Indeed, immunofluorescence microscopy revealed that the majority of BxPC-3 cells strongly expressed the epithelial marker E-cadherin on the cell membrane, whereas only a few cells near the margin of cell clusters expressed the mesenchymal marker vimentin in their cytoplasm; in contrast, MIA PaCa-2 cells were negative for E-cadherin but showed uniform, strong vimentin expression (Fig. 3a). Importantly, this expression pattern was conserved when the cells were grown as xenografts in mice (Fig. 3b). Further, we analyzed PC-3 cell cultures and xenograft tumors and observed that the majority of cells had lost E-cadherin membrane expression; only a subset of tumor cells were E-cadherin positive (Supplement Fig. 2), and the percentage of these epithelial-like cells varied from experiment to experiment. These results formed the initial basis for the hypothesis that a signature of EMT in carcinomas may be linked to sensitivity to FAK inhibitors.

To further explore this concept we tested the efficacy of BI 853520, administered daily per os at a standard dose of 50 mg/kg, in additional models representing several types of adenocarcinomas. We observed a broad range of treatment outcomes (Table 3) and arbitrarily classified these models as “highly sensitive” when the TGI values were ≥89% (tumor regressions were often observed in subsets of animals), as “moderately sensitive” for statistically significant TGI values of 57–83% and as “resistant” when the TGI values (14–45%) were non-significant. Drug sensitivity was observed across multiple cancer types with the exception of colorectal carcinoma, although it must be noted that only two CRC models were included. All highly sensitive models, representing kidney, lung, ovarian, pancreas and prostate adenocarcinomas, were found to lack E-cadherin expression (*n* = 6) or to express low levels of E-cadherin (*n* = 1), defined as membrane staining in <30% of the tumor cells. Among the four moderately sensitive models, three were E-cadherin low/negative (TGI 66, 72, and 83%) and one, the SK-OV-3 ovarian carcinoma model, showing the lowest significant TGI value of 57%, was E-cadherin positive. Of five resistant models, three were E-cadherin positive, one (NCI-H2122, lung carcinoma) stained negative for E-cadherin but is known to harbor a somatic E-cadherin mutation resulting in a deletion of six amino acids, and one was E-cadherin low in the absence of a mutation (SW480 colon carcinoma).

**Table 3:** Efficacy of BI 853520 and expression of E-cadherin and hsa-miR-200c-3p in human adenocarcinoma models

Organ derivation	Cell line	Median TGI (%)	Sensitivity	E-cadherin protein expression	*CDH1* mRNA expression (log 2)	hsa-miR-200c-3p expression (log 2)
Colon/rectum	LoVo	14 (n.s.)	Resistant	Positive	8.80	13.69
	SW480	26 (n.s.)	Resistant	Low	6.63	13.67
Kidney	A-498	93	Highly sensitive	Negative	4.19	8.25
	Caki-1	66	Moderately sensitive	Negative	5.69	n.a.
Lung	Calu-6	102	Highly sensitive	Negative	4.06	7.25
	HCC-461	102	Highly sensitive	Negative	4.49	6.38
	NCI-H2122	45 (n.s.)	Resistant	Negative^a^	8.33	13.56
	A549	15 (n.s.)	Resistant	Positive	9.07	n.a.
Ovary	A2780	89	Highly sensitive	Negative	4.11	n.a.
	SK-OV-3	57	Moderately sensitive	Positive	6.97	10.25
	TOV-21G	93	Highly sensitive	Negative	4.70	7.63
Pancreas	AsPC-1	13 (n.s.)	Resistant	Positive	9.02	13.28
	MIA PaCa-2	104	Highly sensitive	Negative	3.76	7.90
	PANC-1	83	Moderately sensitive	Low	5.56	7.06
Prostate	PC-3	93	Highly sensitive	Low	8.87	6.24
Stomach	Hs 746T	72	Moderately sensitive	Negative	4.04	9.89

Mice bearing subcutaneous tumors were treated with 50 mg/kg BI 853520 once daily per os (seven or ten animals per group). E-cadherin protein expression in control tumors was determined by immunohistochemistry; tumors were classified as “negative” if expression was not detectable, as “low” if less than 30% of cells expressed E-cadherin on their membranes, and as “positive” if 30% or more expressed E-cadherin. Expression of *CDH1* (=gene encoding E-cadherin) mRNA and of hsa-miR-200c-3p microRNA was analyzed using Affymetrix GeneChip Exon 1.0 and Affymetrix GeneChip miRNA 3.0, respectively (2–3 tumors per group)

*n.s.* statistically not significant (*p* > 0.05), *n.a.* data not available

^a^CDH1 mutation in NCI-H2122

### Sensitivity to BI 853520 and EMT

**Fig. 4:**
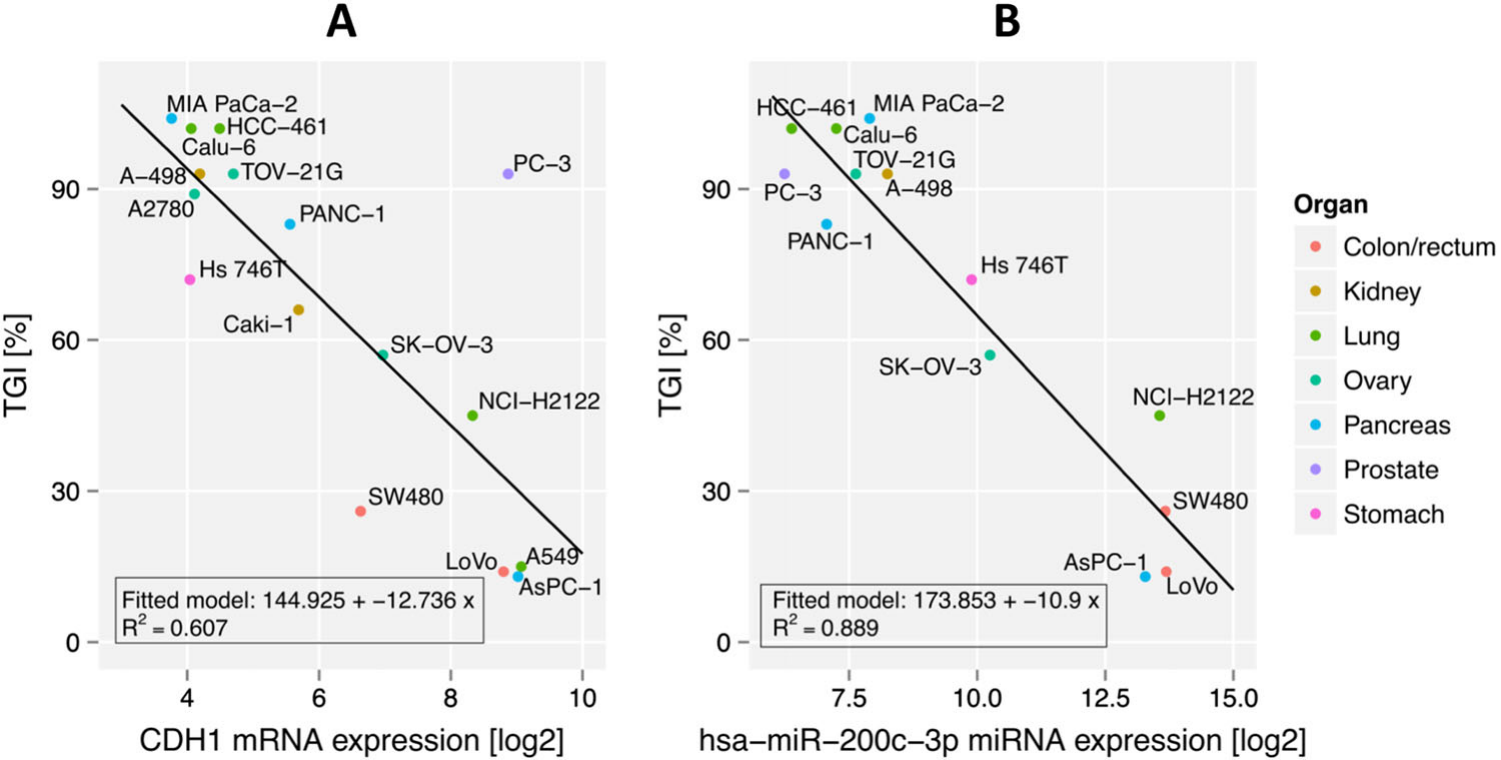
E-cadherin mRNA and hsa-miRNA-200c-3p expression and sensitivity to BI 853520 in adenocarcinoma xenograft models. BI 853520 was dosed at 50 mg/kg daily per os, TGI values represent the median of 7 or 10 animals per group, RNA expression values represent the mean of 2-3 control tumors. **a** E-cadherin mRNA expression (*CDH1*). **b** hsa-miR-200c-3p expression

In order to obtain independent confirmation of the relationship between E-cadherin expression and sensitivity to BI 853520 and move towards a more quantitative correlation we analyzed expression of E-cadherin mRNA by GeneChip analysis (Table 3). In general, we observed concordance between protein and mRNA expression, with the exception of PC-3 tumors which showed high mRNA levels but low protein expression. A remarkable correlation between E-cadherin mRNA expression and TGI was noted (Fig. 4a), with PC-3 tumors as an obvious outlier.

We next attempted to move beyond E-cadherin as a marker for EMT, a process which involves changes in multiple signaling pathways and in the expression of hundreds of genes and can be partial or complete, reversible or irreversible. Expression of eight additional genes associated with EMT was initially analyzed individually (Supplement Tables 2 and 3, and Supplement Fig. 3); a good correlation between tumor sensitivity to BI 853520, and gene expression was observed for MUC13 and POF1B but not for TWIST1. We further performed a comprehensive gene set enrichment analysis and identified several sets that are linked to EMT (Supplement Table 4 and Supplement Fig. 4).

In addition to protein-encoding genes, expression of microRNA genes has been associated with the regulation of EMT in normal and malignant cells^9^. We initially analyzed multiple microRNAs in adenocarcinoma xenograft models, comparing six highly sensitive models to five resistant models. Our results indicate that 57 microRNAs are differentially expressed in those groups (adjusted *p* value ≤0.05 and fold-change ≥2; Supplement Table 5); of these, hsa-miR-200c-3p showed the most pronounced difference: expression was on average 68-fold lower in highly sensitive than in resistant tumors (adjusted *p* value, 1.03e-12). Table 3 and Fig. 4b illustrate the relationship between hsa-miR-200c-3p expression and TGI for all samples analyzed. All highly sensitive tumors expressed low levels of hsa-miR-200c-3p (log2 expression values, 6.24–8.25; *n* = 7); whereas all resistant tumors showed high expression (13.13–13.69; *n* = 4). Moderately sensitive tumors showed low (7.06) to intermediate expression (9.89 and 10.25, respectively).

## Discussion

The human kinome encodes more than 500 structurally related kinases, all characterized by a conserved ATP binding pocket; design of an ATP-competitive inhibitor for a specific kinase deregulated in cancer can thus be a formidable task. Although it has been argued that multi-kinase inhibitors may have therapeutic advantages in view of the fact that multiple signaling pathways are dysregulated in malignant cells and their microenvironment^10^, the promise of precision medicine can only be realized when highly selective inhibitors are used to avoid adverse events due to off-target effects, such as cardiotoxicity, bone marrow suppression or hand-foot syndrome. We have therefore selected BI 853520 for in-depth profiling as a FAK inhibitor, as this compound showed excellent selectivity across a large panel of kinases representing different branches of the kinase tree, and in particular was highly selective against PYK2, FAK´s closest relative in the kinome. Additional evidence supporting a favorable profile of BI 853520 include the high selectivity (1000-fold) for inhibiting proliferation of PC-3 cells in a soft agar matrix, but not in conventional culture. The compound shows a desirable pharmacokinetic profile in mice with good bioavailability, high volume of distribution and adequate half-life, and treatment of mice resulted in long-lasting suppression of target kinase activity in xenografted human tumors at doses and schedules that were well tolerated by the animals upon prolonged treatment. In certain xenograft models, such as the MIA PaCa-2 pancreas adenocarcinoma, this dose level was not only sufficient to fully inhibit tumor growth, but resulted in tumor shrinkage in a subset of animals. BI 853520 is thus a promising compound for further exploration as an anti-cancer agent, and due to its favorable tolerability may be useful for combination with a wide range of established and investigational anti-cancer agents.

Several years after the initiation of the first clinical trial of a FAK inhibitor, the major issue limiting the clinical utility of this class of agents remains the selection of patients most likely to benefit from therapy. As outlined above, clinical trials involving biomarker-based patient selection were initiated (merlin expression, *CDKN2A* mutation; ClinicalTrials.gov identifiers: NCT01870609, NCT01938443, NCT01951690). Our results, based on a broad range of in vivo models, provide the basis for an additional hypothesis, indicating that a mesenchymal tumor phenotype may represent a necessary, although possibly not sufficient, prerequisite for benefit from FAK inhibition. On a cautionary note, these results were obtained using tumors derived from established cancer cell lines that may not fully reflect the genotype and phenotype of the original tumor. Accordingly, a recent study has demonstrated that culture of freshly explanted pancreatic carcinoma cells may result in irreversible EMT^11^. However, there is evidence that EMT indeed occurs in real-world cancers and impacts on the natural history of the disease. For example, immunohistochemical analysis of 329 cases of surgically resected pancreatic adenocarcinomas revealed that 43% of these samples displayed loss of E-cadherin expression, although complete loss was observed in only seven cases; moreover, partial loss of E-cadherin was found to be an independent predictor of adverse outcome^12^. In an independent study of 174 pancreas adenocarcinoma patients, a mesenchymal tumor phenotype (defined as loss of E-cadherin and gain of vimentin expression) was associated with poor prognosis^13^. The EMT phenomenon is likely not limited to adenocarcinomas; for example, a recent study has investigated expression of E-cadherin and vimentin in esophageal squamous cell carcinomas and found evidence for a post-EMT status in 63 out of 105 cases; again, mesenchymal tumor characteristics were associated with poor overall survival^14^.

Although immunohistochemical detection of E-cadherin and vimentin protein expression in tumor samples is technically straightforward, the complexity of cancer genomes on one hand and of the EMT phenomenon on the other may require analysis of additional molecular parameters. Multiple markers of EMT, such as the transcription factors Snail, Twist and ZEB, have been proposed and characterized^15^. In our data set, however, we see the best correlation of response and gene expression for other EMT markers, such as MUC13, GPX8, and POF1B (Supplement Fig. 3). In addition, we performed an unbiased Gene Set Enrichment Analysis and found that top scoring gene sets are enriched for EMT signatures (Supplement Fig. 4).

Moving beyond mRNA-based markers, we analyzed the microRNA gene expression in our xenograft tumor samples. Our results indicate that hsa-miR-200c-3p may represent a useful additional, or alternative, marker for EMT and sensitivity to FAK inhibition. In the majority of our tumor models, low hsa-miR-200c-3p expression correlated with low E-cadherin expression, however, hsa-miR-200c-3p expression data have helped to resolve inconsistencies in several cases. For example, SW480 colon carcinomas were resistant to BI 853520 even though they were classified low for E-cadherin; high hsa-miR-200c-3p expression has clearly identified these tumors as epithelial-like. Similarly, inhibitor-resistant NCI-H2122 tumors were E-cadherin negative upon antibody staining but showed high hsa-miR200c-3p expression; SK-OV-3 tumors, moderately sensitive to BI 853520 in spite of marked E-cadherin expression, showed intermediate levels of hsa-miR-200c-3p expression. Additional studies will be required to demonstrate that quantification of hsa-miR-200c-3p expression in patient-derived tumor samples is technically feasible and to survey the frequency distribution of hsa-miR-200c-3p expression and correlation with E-cadherin expression in various cancer types.

Taken together, our experiments have shown that in vivo efficacy of the highly selective FAK inhibitor BI 853520 in mouse adenocarcinoma models is linked to a mesenchymal tumor phenotype characterized by low E-cadherin mRNA and protein levels and by low expression of miRNA hsa-miR-200c-3p. Ongoing studies are currently assessing the efficacy of BI 853520 in tumor models in combination with established and investigational drug classes. Further, we are currently extending our work to additional types of cancer, including those derived from mesenchymal tissues, and have observed that certain sarcoma models are sensitive to FAK inhibition^16^. Interestingly, a recent study^17^ has demonstrated a significant correlation between resistance to FAK inhibition and E-cadherin expression in vitro in a panel of cell lines derived from mesotheliomas, a mesenchymal tumor type. Meanwhile, clinical development of BI 853520 has been initiated (ClinicalTrials database accession numbers NCT01905111 and NCT01335269); results available to date show that the compound is orally bioavailable, support a once-daily dosing scheme and indicate a favorable safety profile^18,19^.

## Material and methods

### Compound

BI 853520 was synthesized at Boehringer Ingelheim RCV GmbH & Co KG^20^.

### Kinase assays

IC_50_ values on FAK and PYK2 were determined using the DELFIA^®^ format, using kinase proteins obtained from Invitrogen (#PV3832, #PV4567) and biotinylated poly(Glu,Tyr) as substrate. Incubation in the presence of test compound was followed by detection of phosphorylated substrate using streptavidin-coated plates and Eu-N1 anti-phospho-Tyr PT66 antibody (#AD0040, VICTOR™ reader, both Perkin Elmer). Data were analyzed using GraphPad Prism. Inhibitor concentrations were transformed to logarithmic values and raw data were normalized using 100% and blank controls; these values were used to calculate IC_50_ values. Data was fitted by iterative calculation using a sigmoidal curve analysis with variable Hill slope. In independent assays, IC_50_ values were determined using Z'-LYTE^®^ technology, which is based on FRET (Invitrogen/Life Technologies). Selectivity was determined on the Invitrogen kinase panel at 1 µmol/L.

### Cell lines

Cell lines were cultured according to the provider’s instruction and authenticated by STR analysis. A101D, A549, AsPC-1, BxPC-3, Caki-1, Calu-6, Hs 746 T, LoVo, MIA PaCa-2, NCI-H2122, PANC-1, PC-3, SK-OV-3, SW480 and TOV-21G were from the ATCC. A2780 and A-498 were from the ECACC and DSMZ, respectively. HCC-461 was a kind gift from Dr. Heidi Greulich (Dana Farber Cancer Institute).

### Phospho-FAK Y397 assay

PC-3 cells were treated with compounds for 2 h, fixed with 4% formaldehyde in PBS, washed and incubated overnight with a FAK pY397-specific rabbit monoclonal antibody (Invitrogen/BioSource #44-625 G, 1:2 000). Signal was detected by adding goat-anti-rabbit IgG coupled to horse radish peroxidase (Dako #P0448, 1:750) and TMB substrate solution. Data analysis was performed as described above. Normalized raw data were used to calculate EC_50_ values.

### Proliferation assays

PC-3 cells were incubated with compounds for 5 days at 37 °C/5% CO_2_, stained with alamarBlue^®^ and fluorescence was determined (544 nm/590 nm). Data were analyzed as described above.

Drug sensitivity of a panel of human cancer cell lines was tested at Eurofins Panlabs (Bothell, WA, USA). Cells were treated for 72 h, fixed and stained with diaminophenylindole to visualize nuclei and the fluorescence intensity of each well was measured (GE Healthcare IN Cell Analyzer 1000). Relative cell proliferation in compound-treated cultures (signal as percent of control, “POC”) was calculated as follows: POC (*t* = 72 h) = 100 × fluorescence (compound wells) / fluorescence (control wells). In addition, for each compound-treated culture, the cell count after incubation for 72 h (POC [*t* = 72 h]) was related to the count at start of treatment (POC [*t* = 0 h]): POC [*t* = 0 h] = 100 × fluorescence at *t* = 0 [control wells]/fluorescence at *t* = 72 h [control wells]). To calculate concentration–response curves, POC data were analyzed using a four-parameter log-logistic function without upper or lower limitation.

### Clonogenic assays

Clonogenic assays were performed using agarose-containing media. Cells were incubated at 37 °C and 5% CO_2_ for 14 days. alamarBlue^®^ was used to detect colonies of viable cells (530 nm/590 nm). Data analysis was performed as described above.

### Animal Studies

Mice were kept and experiments performed in an AAALAC-accredited SPF facility according to institutional and governmental guidelines; study protocols were reviewed and approved by the institutional Ethics Committee and the responsible governmental committee.

Mice were housed in Macrolon^®^ type II or III cages, soiled with bedding, in groups of 3 or 7–10 at 21.5 ± 1.5 °C temperature and 55 ± 10% humidity in a 12 h/12 h light/dark cycle. Standardized diet and autoclaved tap water were provided ad libitum. Subcutaneous microchips implanted under isoflurane anesthesia were used for identification.

For pharmacokinetic studies, single doses of BI 853520 were administered to female BomTac:NMRI-*Foxn1*^nu^ mice (5 mg/kg *i.v*.; dissolved in 25% HP-ß-CD at pH 6.0, or 50 mg/kg *p.o*., suspended in 1 mol/L HCl and diluted with 0.5% Natrosol^®^) and plasma samples were taken after 5, 30 min, 1, 6, 24 h for *i.v.*, and 30 min, 1, 2, 6, 24 h for *p.o.* studies (*n* = 3).

For PK-PD studies BomTac:NMRI-*Foxn1*^nu^ mice xenotransplanted with 5 × 10^6^ PC-3 cells were orally treated with 50 mg/kg BI 853520 or with the vehicle on four consecutive days (*n* = 3). Tumors were taken after 2, 4, 6, 24 h or 48 h.

For efficacy studies, 1 × 10^7^ MIA PaCa-2 or 1 × 10^6^ BxPC-3 cells (with Matrigel^®^ 50:50) were injected subcutaneously into the right flank of female BomTac:NMRI-*Foxn1*^nu^ mice (age 6 weeks, 24-30 g; Taconic, Denmark). These two and the following tumor models were established from adherently grown cells which were either subcutaneously injected in PBS (LoVo, SW480, A-498, Calu-6, NCI-H2122, A549, SK-OV-3, TOV-21G, AsPC-1, PC-3) or were injected with Matrigel (HCC-461, A2780, PANC-1 and Hs 746 T). For the Caki-1 tumor model tumor fragments were passaged in mice. Mice were randomized according to tumor volume when tumors had reached diameters of 6–8 mm. BI 853520 suspended in 1 mol/L HCl and diluted with 0.5% Natrosol^®^ was administered intragastrically at 10 ml/kg once daily (*n* = 7 for BxPC-3 and *n* = 10 for MIA PaCa-2). Natrosol-treated animals served as vehicle controls (*n* = 10). Tumor diameters were measured three times weekly using a caliper and volumes were calculated according to the formula “tumor volume = length × diameter^2^ × π/6”. Mice were inspected daily for abnormalities and body weight was determined three times weekly. Animals were euthanized at the end of the study or prematurely, based on criteria including weight loss exceeding 20%, tumor necrosis or tumor volumes exceeding 1500 mm^3^.

Statistical evaluation was performed using SAS 8.2 (SAS Institute, Cary, NC, USA) and Proc StatXact (Cytel Software, Cambridge, MA, USA). Tumor growth inhibition (TGI) from day 1 until day *d* was calculated as follows:

$${\mathrm {TGI}} = 100 \times \frac{{\left( {C_{\mathrm d} - C_1} \right) - \left( {T_{\mathrm d} - T_1} \right)}}{{\left( {C_{\mathrm d} - C_1} \right)}}$$

where *T*_*d*_ and *C*_*d*_ denote the median of the tumor volume in the treatment group and the control group at day *d*, respectively. A one-sided increasing Wilcoxon test was applied to compare each experimental group with the vehicle control, the *p* values were adjusted for multiple comparisons according to Bonferroni–Holm. The level of significance was fixed at *α* = 5%. An adjusted *p* value of <0.05 was considered to show a significant difference between groups.

### Analysis of E-cadherin expression

Paraffin sections were analyzed using the avidin–biotin complex immunoperoxidase procedure as described^21^. The following antibodies were used: HECD1 against E-cadherin (Abcam ab1416, Cambridge, UK), V9 against vimentin (V6630, Sigma-Aldrich, St. Louis, MO). Epitope retrieval was carried out in citrate buffer. The M.O.M. kit was used to block non-specific binding to mouse tissues and the Vectastin ABC Elite kit (Vector Labs, #PK-6200) as a detection system followed by DAB and hematoxylin counterstaining. Cultured cells grown on chamber slides to near confluency were fixed in acetone/methanol and stained with the antibodies listed above. Binding was detected using Alexa Fluor^®^ 488-conjugated goat-anti-mouse antibody (Molecular Probes, Invitrogen) followed by DNA staining with propidium iodide.

### Analysis of plasma and tumor samples

Plasma and homogenized tumor samples were extracted and analyzed by LC-MS. Detection was at unit resolution with a MRM 589.2/474.8. All calibration and QC samples were with ±20% (*p.o*.) and ±30% (*i.v*.) of the nominal value in the linear range of the calibration fitted with a linear regression and 1/x^2^ weighting.

### mRNA expression profiling

RNA was extracted from cryo-sections of xenograft tumors using TRIzol^®^ (Life Technologies) and purified using a miRNeasy Mini kit (Qiagen). The protocol “Affymetrix GeneChip^®^ Whole Transcript Sense Target Labeling Assay” with 300 ng of total RNA was used for cRNA synthesis. Double-stranded cDNA was generated with random hexamers tagged with a T7 RNA promoter sequence and subsequently used as a template for in vitro transcription and amplification with T7 RNA polymerase with dUTP incorporated. Fragmented and labeled single-stranded cDNA was hybridized to Affymetrix GeneChip^®^ Exon 1.0 arrays and scanned using a GeneChip^®^ Scanner 3000 7G. CEL files were produced using GeneChip Operating Software v. 1.3 and normalized using the RMA method as implemented in Bioconductor version 2.14. RMA was used with a RefSeq based custom CDF (version 18) from the brainarray website (http://brainarray.mbni.med.umich.edu/Brainarray/Database/CustomCDF/CDF_download.asp).

### MicroRNA expression profiling

One microgram of total RNA was used for labeling with FlashTag^®^ Biotin HSR Kit (Genisphare) and subsequently hybridized to Affymetrix GeneChip^®^ miRNA Arrays (version 3.0). After scanning, CEL-files were processed using either the Affymetrix MiRNA QC TOOL or the Transcriptome Analysis Console, and subsequently quantile-normalized and log2-transformed.

### Differential gene expression analysis for mRNA/miRNA data

Normalized mRNA/miRNA expression data was loaded into R 3.1.1^22^ and differentially expressed probes were detected using limma 3.20.9 from Bioconductor version 2.14^23^.

### Data availability

Gene chip data was submitted to the Gene Expression Omnibus (accession number GSE 109304), database repository.

## Electronic supplementary material

Supplement Information Summary

Supplement Figure 1

Supplement Figure 2

Supplement Figure 3

Supplement Figure 4

Supplement Table 1

Supplement Table 2

Supplement Table 3

Supplement Table 4

Supplement Table 5

Supplement Table 6
