# Supplementary material for: Efficacy of the highly selective focal adhesion kinase inhibitor BI 853520 in adenocarcinoma xenograft models is linked to a mesenchymal tumor phenotype
Source: Oncogenesis. 2018 Feb 23;7(2):21. doi: 10.1038/s41389-018-0032-z (PMC5833389; doi:10.1038/s41389-018-0032-z)
Supplement: Supplementary file 1 — Supplement Information Summary [file 41389_2018_32_MOESM1_ESM.docx]

**Supplement Figure 1. Efficacy of BI 853520 in pancreas adenocarcinoma xenograft model MIA‑PaCa-2 in nude mice.**
Animals were treated with 12.5 or 25 mg/kg BI 853520 daily *per os*. Data represent median tumor volumes (left graph) and individual as well as median values (right graph) at the given day, 10 animals/group.

File format: pptx

**Supplement Figure 2:** **E-cadherin and vimentin expression in PC-3 xenografts**

Tumor xenografts from PC-3 cells were fixed and processed for immunohistochemistry as described (see Material & Methods). Expression of E-cadherin was observed in small subsets of tumor cells only, whereas vimentin expressed homogeneously across the tumors. Size bars represent 60µm.

File format: pptx

**Supplement Figure 3: mRNA expression and sensitivity to BI 853520 in adenocarcinoma xenograft models.**

**A)** TWIST1 (mesenchymal marker)

**B)** CDH2 (mesenchymal marker)

**C)** FN1 (mesenchymal marker)

**D)** SNAI1 (mesenchymal marker)

**E)** VIM (mesenchymal marker)

**F)** MUC13 (epithelial marker)

**G)** GPX8 (mesenchymal marker)

**H)** POF1B (epithelial marker)

TGI values as in Fig. 4.

File format: pptx

**Supplementary Figure 4: Gene set enrichment analysis in xenografts based on mRNA gene expression.
A)** Gene set showing high expression (red colors in heatmap) in the sensitive cell lines (yellow block in the heatmap).
**B)** Gene set with high expression in the resistant cell lines (grey block in the heatmap).

File format: pptx

**Supplement Table 1: Dose-response curves for selected cell lines**

A large panel of cell lines was tested for anti-proliferative activity of BI 853520. EC_50_ values were either calculated or estimated when the value at the highest concentration (10 µM) was considered to be an outlier and excluded.

File format: xlsx

**Supplement Table 2: Differential expression analysis from GeneChip® Human Exon 1.0 ST Array data.**

This table shows results from the strict cutoff (sensitive TGI ≥ 89; resistant TGI ≤ 50).

File format: xlsx

**Supplementary Table 3: Extended version of Table 3 showing median gene expression values for the following additional genes: VIM, TWIST1, GPX8, CDH2, FN1, SNAI1, POF1B, and MUC13**.

This data is used for the plots in Supplementary Figures 3.

File format: xlsx

**Supplement Table 4: Gene set enrichment analysis listing C2 (curated genes sets) MsigDB gene sets enriched in sensitive and resistant cell lines**.

The list is manually pre-filtered on significant gene sets (FDR q-value ≤ 0.05) related to EMT. All models satisfying the strict cutoff (sensitive TGI ≥ 89; resistant TGI ≤ 50) were used in the analysis.

File format: xlsx

**SupplementTable 5: Differential expression analysis from GeneChip® miRNA Array 3.0 data.**

This table shows results from the strict cutoff (sensitive TGI ≥ 89; resistant TGI ≤ 50). The table is pre-filtered by significantly differentially expressed miRNA genes (abs(log2FC)≥1, adjusted p value ≤ 0.05).

File format: xlsx

**Supplementary Table 6: Overview of data sets used in the different analysis and data availabilities.**

File format: xlsx
