# Supplementary figures and images for: Efficacy of the highly selective focal adhesion kinase inhibitor BI 853520 in adenocarcinoma xenograft models is linked to a mesenchymal tumor phenotype
Source: Oncogenesis. 2018 Feb 23;7(2):21. doi: 10.1038/s41389-018-0032-z (PMC5833389; doi:10.1038/s41389-018-0032-z)

## Slide 1
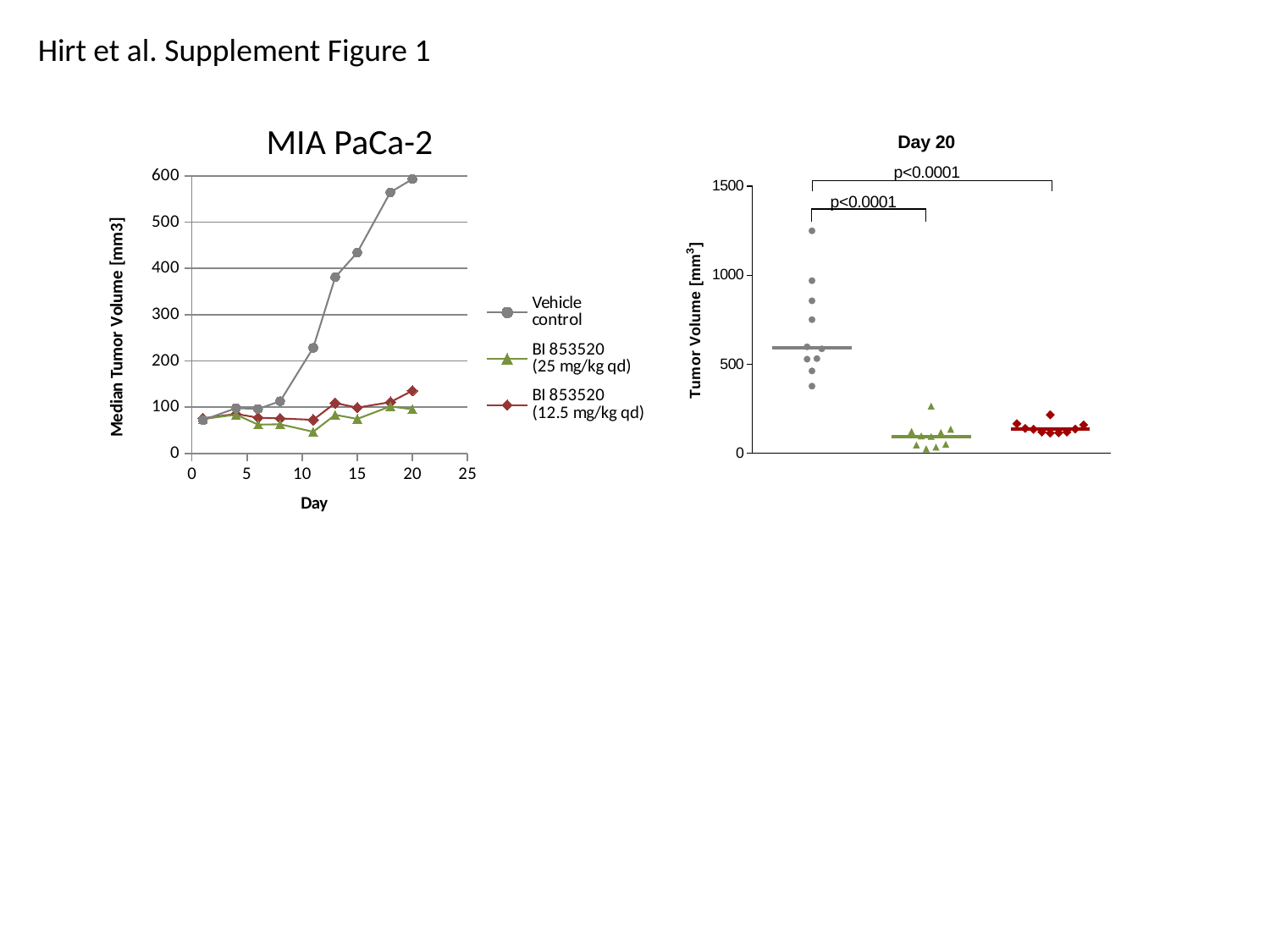

Hirt et al. Supplement Figure 1
MIA PaCa-2
### Chart
| Category | | | |
|---|---|---|---|

Supplement: Supplementary file 2 — Supplement Figure 1 [file 41389_2018_32_MOESM2_ESM.pptx]

## Slide 1
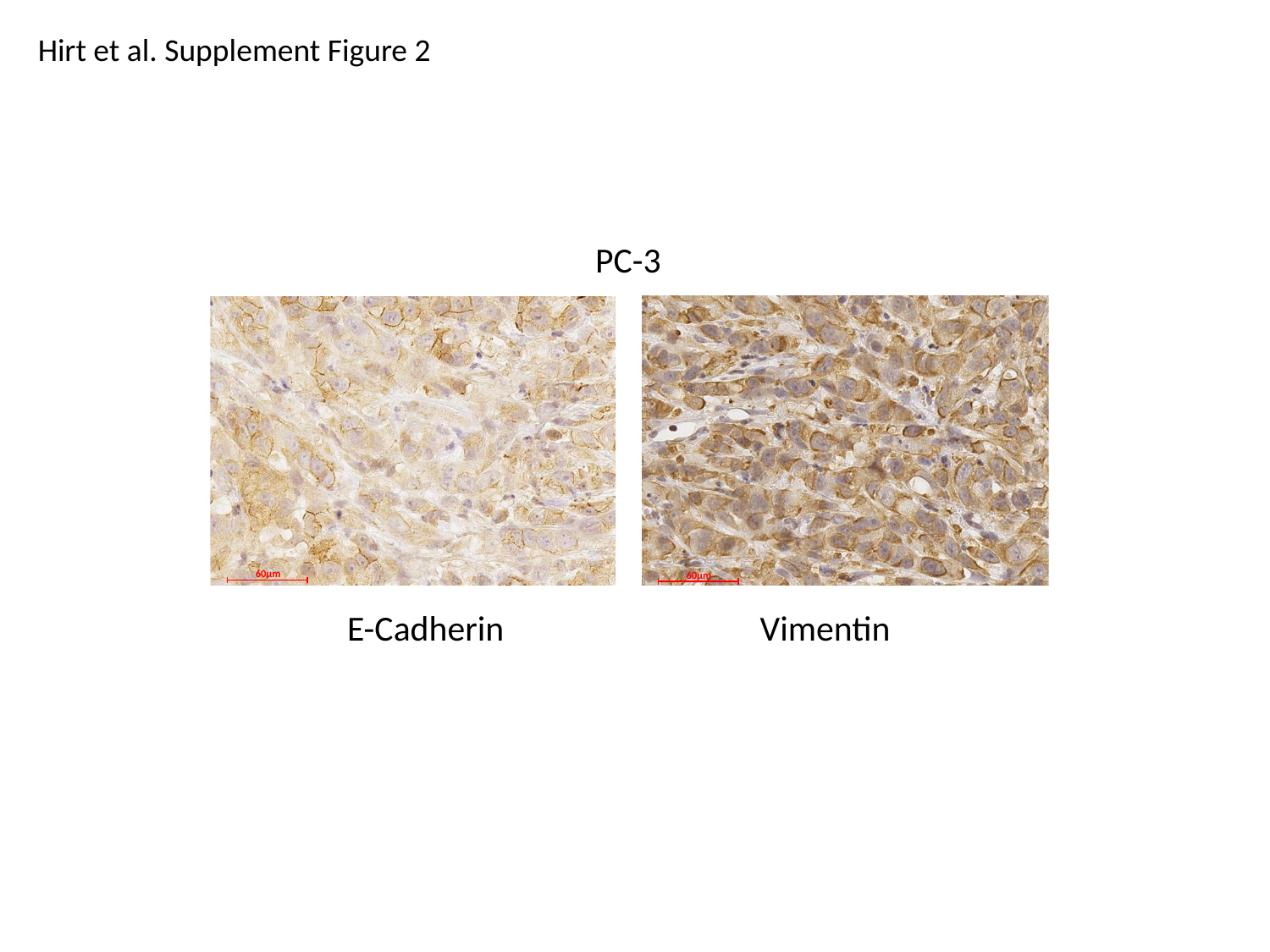

Hirt et al. Supplement Figure 2
PC-3
60µm
60µm
E-Cadherin
Vimentin

Supplement: Supplementary file 3 — Supplement Figure 2 [file 41389_2018_32_MOESM3_ESM.pptx]

## Slide 1
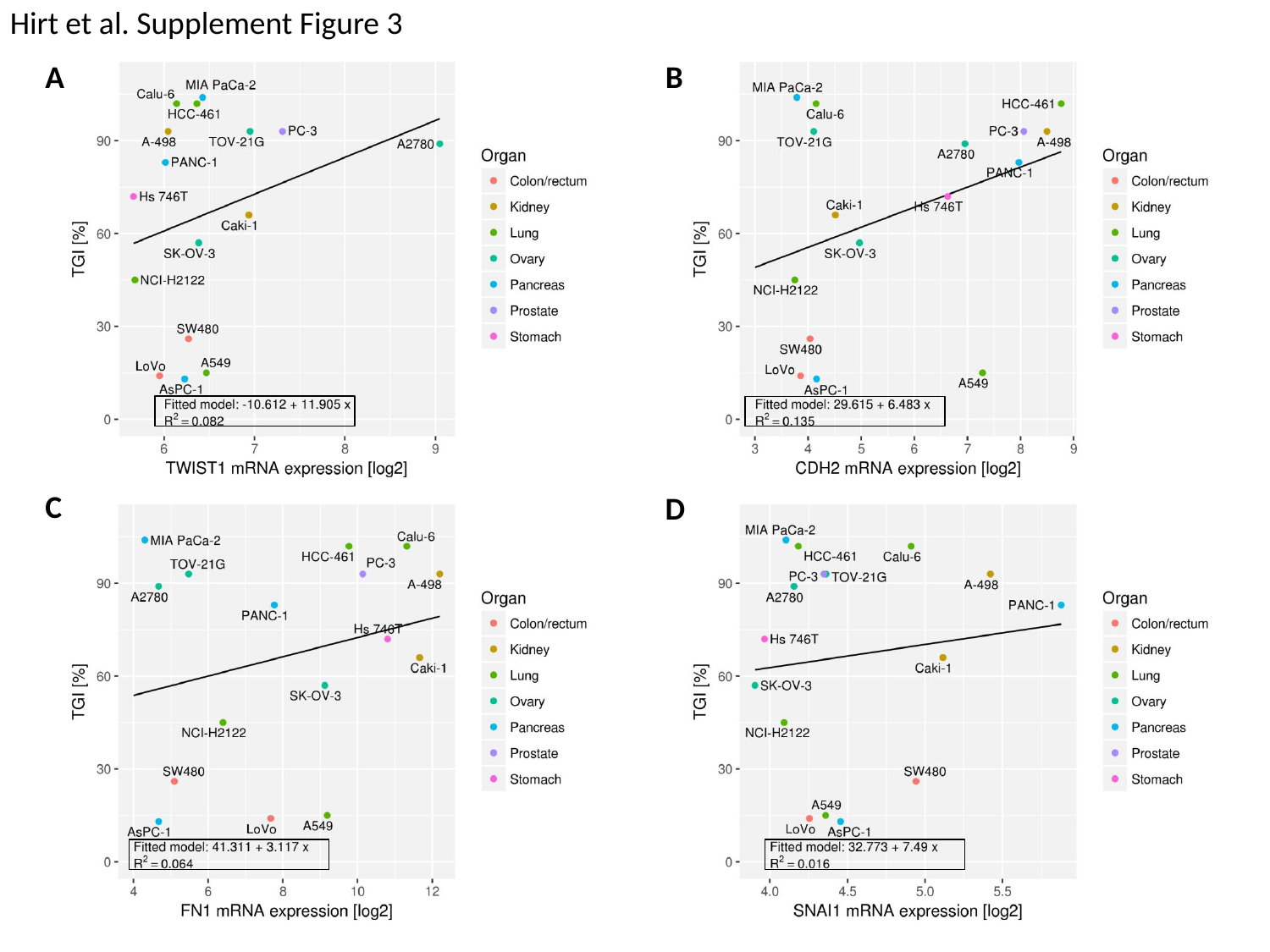

Hirt et al. Supplement Figure 3
A
B
C
D

## Slide 2
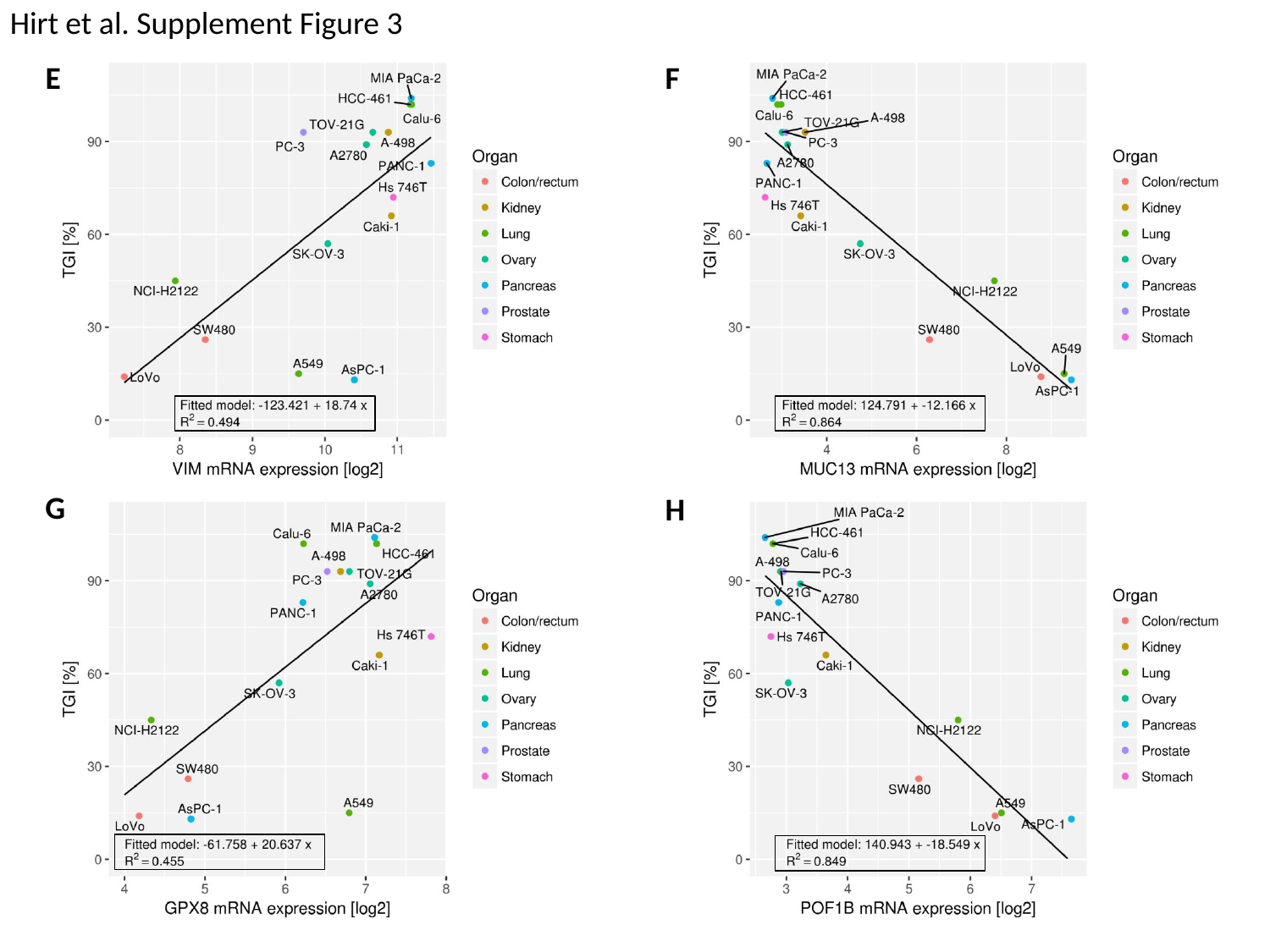

Hirt et al. Supplement Figure 3
E
F
G
H

Supplement: Supplementary file 4 — Supplement Figure 3 [file 41389_2018_32_MOESM4_ESM.pptx]

## Slide 1
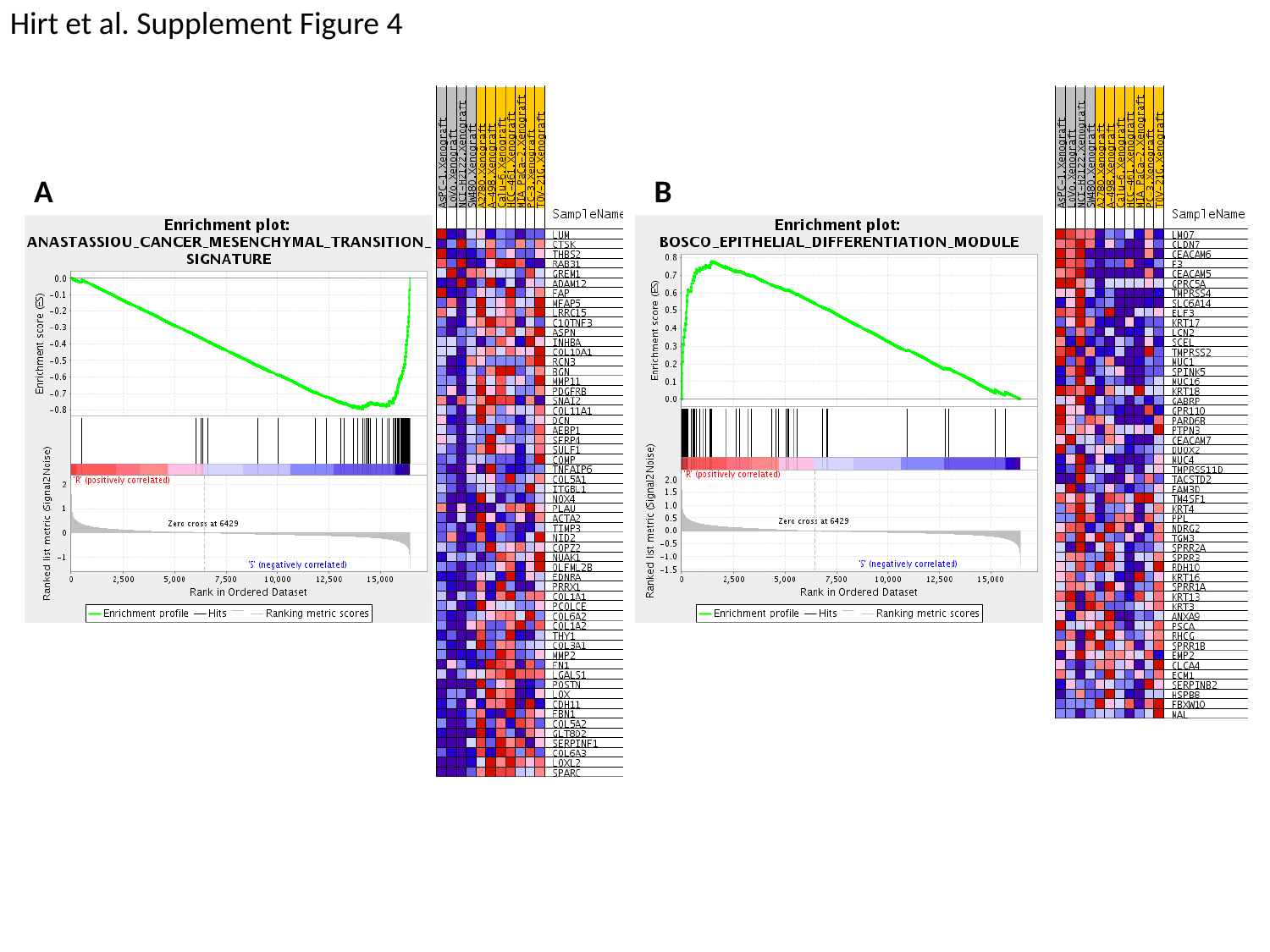

Hirt et al. Supplement Figure 4
A
B

Supplement: Supplementary file 5 — Supplement Figure 4 [file 41389_2018_32_MOESM5_ESM.pptx]
